# Editorial: Mechanisms of Fluorescent Proteins

**DOI:** 10.3389/fmolb.2021.701523

**Published:** 2021-05-26

**Authors:** Chong Fang, Mikhail Drobizhev, Ho Leung Ng, Periklis Pantazis

**Affiliations:** ^1^ Department of Chemistry, Oregon State University, Corvallis, OR, United States; ^2^ Department of Cell Biology and Neuroscience, Montana State University, Bozeman, MT, United States; ^3^ Department of Biochemistry and Molecular Biophysics, Kansas State University, Manhattan, KS, United States; ^4^ Department of Bioengineering, Imperial College London, London, United Kingdom

**Keywords:** genetically encoded markers, photophysics and photochemistry, chromophore structure and dynamics, bioimaging and engineering, molecular machineries, noncanonical amino acid, ultrafast spectroscopy

**Editorial on the Research Topic**

**Mechanisms of Fluorescent Proteins**

This collection of papers for the Research Topic “Mechanisms of Fluorescent Proteins” (FPs) samples a broad range of research on physical mechanisms, applications, and molecular engineering strategies. The papers demonstrate a combination of experimental and computational approaches and are of broad interest to researchers working on FPs, microscopy, and spectroscopy.

In bioimaging with FPs, increasing the penetration depth and decreasing unwanted scattering are desirable, which have motivated efforts for engineered FPs with redder emission and higher brightness (Subach and Verkhusha, 2012; Dedecker et al., 2013). In a combined experimental and computational work (Gorbachev et al., 2020), the green/red photoconversion of EGFP with reducing agents was investigated and a novel green-emitting state only present under low-oxygen conditions was identified. Following photoconversion, the distinct orange and red-emitting forms (565 and 600 nm emission maxima) differ from the reported red-emitting form (607 nm emission) via oxidative reaction. This work showcases a complex interplay between the chromophore and protein environment, generating a neutral quinoid-like green-emitting chromophore (525 nm emission) as an intermediate. This step leads to a zwitterionic form of the photoexcited chromophore via charge transfer that bifurcates into the orange and red-emitting forms. Such a general oxidative mechanism enriches the FP application toolset (Bourgeois and Adam, 2012; Jung, 2012; Krueger et al., 2020; Nasu et al., 2021) with additional tunable “knobs” of oxygen levels and redox-active compounds to control photoconversion and achieve redder emission.

To brighten the generally dim red and far-red FPs, a systematic study of nonradiative relaxation in red FPs (RFPs) (Drobizhev et al., 2021) reveals a dominant role of the twisted intramolecular charge transfer mechanism over the energy gap law. This work substantiates local electrical field control of fluorescence quantum yield (FQY) of RFPs. Aided by one- and two-photon absorption spectroscopy and quantum calculations of seven different RFPs with the same chromophore structure, a spectroscopic method of evaluating local electric fields (amplitude and direction in *E*_*x*_ and *E*_*y*_) at the protein chromophore enables separation of contributing factors to the nonradiative relaxation rate. A small range of positive or negative values for *E*_*x*_ and *E*_*y*_ (–10 to +10 MV/cm) was revealed to facilitate both a red-shifted absorption and a high FQY, providing rational design principles for site-specific mutagenesis using RFP scaffold like DsRed.

On a fundamental level, the fluorescence mechanism via excited-state proton transfer (Chattoraj et al., 1996; Fang et al., 2009; Tonge and Meech, 2009; Fang and Tang, 2020) is elucidated further by a computational study (Coppola et al., 2020) on the complex hydrogen (H)-bond equilibrium dynamics for neutral, intermediate, and anionic chromophore forms inside GFP. An accurate hybrid QM/MM simulation of the entire protein was performed to enable the intricate correlation between chromophore site-specific single H-bond interactions and the chromophore cavity volume, and noncovalent interactions with distant residues on opposite sides of the pocket. This work showcases the power of *ab initio* molecular dynamics simulations in hybrid form with density functional theory (DFT) to bridge local and larger-scale effects in FPs.

Most of FPs contain a π-conjugated chromophore, *p*-hydroxybenzylidene-imidazolinone (HBDI). Typical examples are GFP from *Aequorea victoria*, and DsRed from *Discosoma* sp. wherein the HBDI chromophore is amended by an acylimine tail that lengthens π-conjugation and red-shifts the absorption. Targeted engineering for brighter variants was typically driven by linear, one-photon absorption and fluorescence. With the advent of two-photon laser microscopy, the need for bright and photostable FPs has increased. Yet two-photon absorption obeys different quantum-mechanical rules compared with one-photon absorption. Consequently, the brightest FPs with canonical chromophores for one-photon imaging are not necessarily optimized for two-photon excitation. The two-photon cross-sections of ten non-canonical chromophores (nCCs), inserting substituted tyrosines into the DsRed scaffold, were calculated using QM/MM schemes with polarizable embedding and external effective field correction (Rossano-Tapia et al., 2020). Although none of the model proteins shows a two-photon cross-section larger than DsRed (List et al., 2016), the work helps to understand structure-function relationships and design better two-photon-absorbing FPs.

Three other FPs with nCCs containing Cl-, Br-, and nitro-substituted tyrosine were prepared from sfGFP scaffold and studied using a combination of femtosecond transient absorption and stimulated Raman spectroscopy (FSRS) (Oscar et al., 2020). The FSRS measurements were supported by DFT calculations of vibration normal modes for accurate assignments. The high spectral and temporal resolution obtained by FSRS and transient absorption allowed delineation of the chromophore protonation state and isomeric structure. Longer vibrational relaxation times in the excited state of Cl-sfGFP (4 ps) and Br-sfGFP (11 ps), compared with the parent sfGFP (1.2 ps), were correlated with the increased FQY. Moreover, FPs with halogenated chromophores exhibit advantageous redshifts in their absorption and emission spectra, rendering them great candidates for bioimaging applications (Pantazis and Supatto, 2014).

Among the most exciting applications of FPs is their use in single-molecule and super-resolution fluorescence microscopy. Such applications necessitate FPs with superior properties such as high FQY, fraction of time remaining fluorescent, outstanding photostability, and structural stability (Bourgeois and Adam, 2012; Nienhaus and Nienhaus, 2016; Woodhouse et al., 2020). In this issue, a bilirubin-activated photoswitching protein called eUnaG was developed with the highest bulk fluorescence to date, comparable to organic dyes (Ko et al., 2021). The superior performance of eUnaG is primarily due to its increased stability, leading to reduced aggregation and fewer labeling artifacts. eUnaG promises to support state-of-the-art performance for high-resolution microscopy.

From this exciting line of inquiries decoding fluorescence mechanisms of FPs, particularly targeting redder and brighter emissions, we foresee the interdisciplinary spectroscopy, microscopy, theoretical, and computational communities to continue joining forces to paint a comprehensive portrait of FPs and implement these molecular machines in ever-expanding applications. Nature has evolved FPs for millions of years, while GFP has revolutionized molecular and cellular biology just for several decades (Shimomura et al., 1962; Chalfie et al., 1994; Tsien, 1998). Much remains to be learned and developed, and we hope this special Research Topic in *Front. Mol. Biosci.* (https://www.frontiersin.org/research-topics/10542/mechanisms-of-fluorescent-proteins#articles) has captured the essence of this field and will inspire future innovations and breakthroughs in both the understanding and applications of FPs.
